# Effect of pre-operatory low-level laser therapy on pain, swelling, and trismus associated with third-molar surgery

**DOI:** 10.4317/medoral.21398

**Published:** 2017-06-04

**Authors:** Morena Petrini, Maurizio Ferrante, Paolo Trentini, Giorgio Perfetti, Giuseppe Spoto

**Affiliations:** 1 DDS, Ph.D, Oral Surgery Specialist, Dental Materials and Medical Chemistry Unit, Department of Medical, Oral and Biotechnological Sciences, University of Chieti; 2 DDS, Oral Surgery Specialist, Dental Materials and Medical Chemistry Unit, Department of Medical, Oral and Biotechnological Sciences, University of Chieti; 3 MD, Dental Materials and Medical Chemistry Unit, Department of Medical, Oral and Biotechnological Sciences, University of Chieti; 4 Full Professor of Oral Surgery, Oral Surgery Unit, Department of Medical, Oral and Biotechnological Sciences, University of Chieti; 5 Full Professor of Dental Materials, Dental Materials and Medical Chemistry Unit, Department of Medical, Oral and Biotechnological Sciences, University of Chieti

## Abstract

**Background:**

The extraction of impacted third molars is commonly associated to pain, edema, trismus, limited jaw opening and movements. The aim of this retrospective study is to verify if pre-surgical low-level laser therapy (LLLT) associated with the extraction of impacted lower third molars could add benefits to the postoperative symptoms respect LLLT performed only after surgery.

**Material and Methods:**

Data from 45 patients subjected to a surgical extraction of lower third molars were pooled and divided into three groups. Patients that received only routine management were inserted in the control group. Group 1, were patients that received LLLT immediately after surgery and at 24 hours. In group 2 were included patients treated with LLLT immediately before the extraction and immediately after the end of the procedure. Data were analyzed using linear regression and descriptive statistics.

**Results:**

Both laser-treated groups were characterized by minor events of post-surgery complications of pain, edema, trismus. The use of NSAIDs in the first 24 hours was significantly inferior in Group 2.

**Conclusions:**

Pre-surgical LLLT treatment seems to increase the analgesic effect of LLLT. However, trismus and edema were reduced in both laser treated groups, independently from the period of irradiation.

** Key words:**Laser, LLLT, pain, surgery, extraction, third molars, wisdom teeth.

## Introduction

The extraction of impacted third molars is commonly associated to postsurgical transient complications of various intensity, like pain, edema, trismus, limited jaw opening and movements (1).

Traditionally these symptoms are treated by the use of glucocorticoids and NSAIDs, but their use should be limited especially in those patients with such disorders or those that already undergone extensive pharmacologic treatments (2). Recent advancements in medicine have permitted the rapid development of light emitting devices for the control of pain and infections (3); one of these is laser, in particular low-level laser therapy (LLLT) (4).

LLLT is able to modulate the inflammatory process without adverse effects, by reducing pain, swelling, and promoting the repair of damaged tissues (5).

Food and Drug Administration has approved the use of LLLT for pain relief in carpal tunnel syndrome since 2002 (6). Since then many data have been published and the use of LLLT for pain treatment developed in all medical fields (7-10). In a previous study, we have shown that postoperative LLLT by the use of a 980 nm diode laser was effective in the reduction of symptoms of pain, edema and trismus associated with third molars extraction (11). However literature shows conflicting opinions about the effects of LLLT: a possible explanation is the fundamental importance of such parameters like the wavelength, the fluence, the power density, the pulse structure and the irradiation time. Consequently, many results published in literature are negatives due to an incorrect chose of the light source or the best protocol (12).

He *et al.* in a systematic review and meta-analysis demonstrated that LLLT was effective in reducing pain, trismus, and swelling after mandibular third molar surgery (13). The heterogeneity of results in literature is probably a consequence of the various protocols and outcomes assessment and risk of bias of the different trials.

Moreover all studies in literature analyzed the effect of LLLT performed only after the third molars extraction, and actually no studies results about the effects of laser biostimulation also before the surgery.

Many studies have highlighted the importance of preoperative anti-inflammatory treatments in reducing postoperative complications (14). Neĭmark *et al.* have found that preoperative low-intensity laser therapy reduced the number of postoperative inflammatory complications, hospital stay, severity of postoperative period in patients with benign prostatic hyperplasia (15). The aim of this retrospective study is to verify the better peri-operatory protocol that is associated with minor complications, after the removal of impacted third molars.

## Material and Methods

In this retrospective study, data from 45 patients subjected to the surgical extraction of lower third molar over the period of five years from September 2010 to October 2015 in the Dental Clinique of the University of Chieti, Italy, were selected, pooled and analyzed. The protocol of this study has been approved by the Local Ethical Committee. Criteria for exclusion included: systemic diseases, local and purulent infections, blood dyscrasia, previous or present gastric ulcers, heart disease, known hypersensitivities, allergies, or idiosyncratic reactions to any study medications, pregnancy and lactation. In addition, patients who had taken analgesics or anti-inflammatory drugs within 24 hours before surgery were not included in the study. The degree of surgical difficulty was assessed on pre-surgical orthopantomography using Pell-Gregory criteria: only III B and III C teeth were included in the study (16). Only extractions that included open flap, odontotomy and osteotomy were included in the study.

- Description of the standardized surgical procedure

All patients before extraction had signed the informed consent and underwent routine pre-operatory management that consisted in the disinfection of the surgical site with 10% povidone-iodine solution (Betadine, Meda Pharma spa, Italy), alveolar nerve block by means of mepivacaine (3%) without epinephrine and infiltration of buccal soft tissues of the same local anesthetic (2%) with 1:100.000 epinephrine (Scandonest, Septodont, France).

Postoperatively, all patients were instructed to apply ice packs directly over the masseteric region on the operated side intermittently (10 minutes intervals) and to assume Ketoprofen, 80 mg orally (Oki, Dompè, Italy), only if was necessary and to record the quantity used in the first 24 hours. Chlorhexidine gluconate mouthwash was prescribed 3 times/day starting from the first day after the surgery until the suture removal (7 days after the extraction). The antibiotic treatment was not prescribed.

- Data collection

Only data from patients treated by the same surgeon were included in the study. Basing on pre- and post-operatory management, data were pooled and allocated into three groups for analysis and statistics.

Controls: patients treated only with routine management.

Group 1: patient subjected to laser irradiation within 10 minutes of completion of the extraction and at 24 h + routine management.

Group 2: patient subjected to the laser irradiation 10 minutes before the start of the surgery and also within 10 minutes of completion of the procedure with the same methodology + routine management.

In all cases the operator who had performed the LLLT in all patients of group test was different from the surgeon; another operator had carried out the measurements.

- LLLT protocol

For treatment, a diode laser device (model: G-Laser 25 Galbiati, Italy) with a continuous wavelength of 980 nm and a 600-µm handpiece was used. Laser energy was applied at 300 mW (0.3 W) for a total of 180 s, 60 s for each point, 0.3 W×180 s=54 J (11). Each LLLT session consisted of an intraoral and an extraoral phase (11).

**Figure 1:**
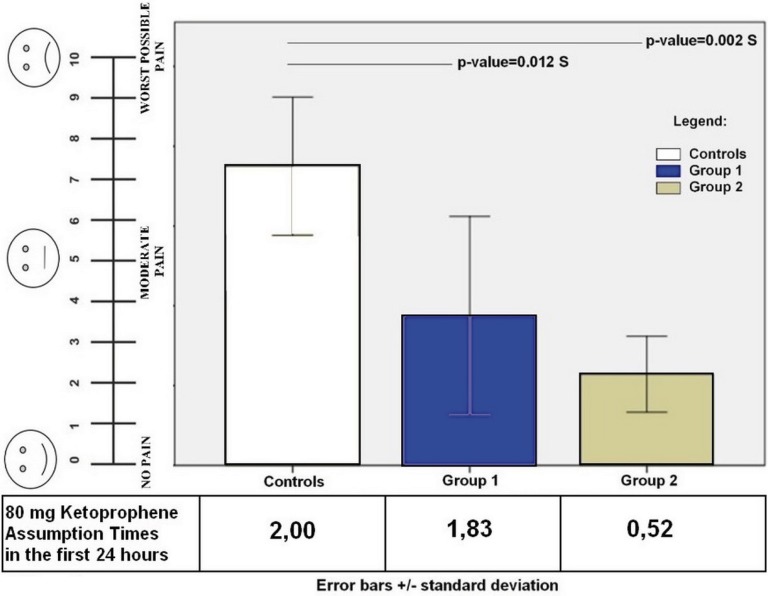
Shows the visual analog scale (VAS) of 10 point for pain measurement and the quantity of Ketoprofen assumption. The level of significance was set at 0.05.

In particular, laser was applied 60 s extraorally at 1 cm from the skin over the area of the masseter, in the side that underwent surgery (Fig. 1A). Then laser was applied intraorally: 60 s on the lingual side of the alveolus of the teeth to be removed and other 60 s on the vestibular wall. The laser was used with circular movements and maintaining a constant distance of 1 cm from gingiva (Fig. 1D).

- Evaluation of postoperative complications

The pain referred by the patients (Fig. 1C) at 24 hours and after 7 days was recorded through a visual analogue scale (VAS). Accordingly, pain was recorded as “0-no pain” (patient experiences no discomfort) to “10-extreme pain” (very noticeable pain which disturbs the patient’s daily routine).

**Figure 2:**
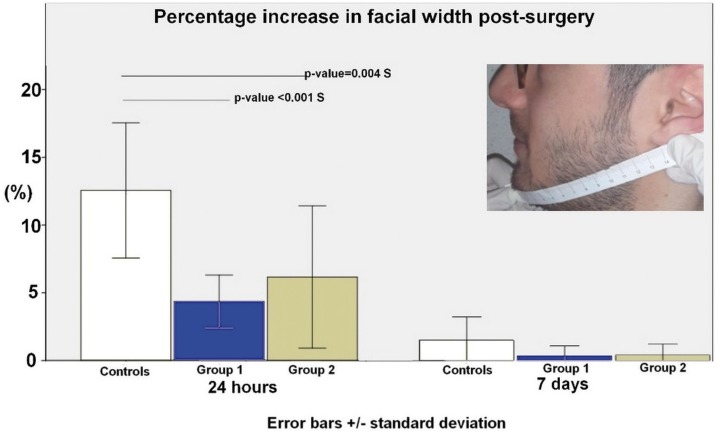
Shows the percentage (%) of increase in facial width after surgery.

The facial swelling was evaluated measuring the distance between the tip of the chin and the lower part of the auricle lobe (Fig. 2). The measurements were carried out just before the surgery and at post-operative days 1 and 7. Postoperative swelling was expressed as a percentage increase in facial width.

**Figure 3:**
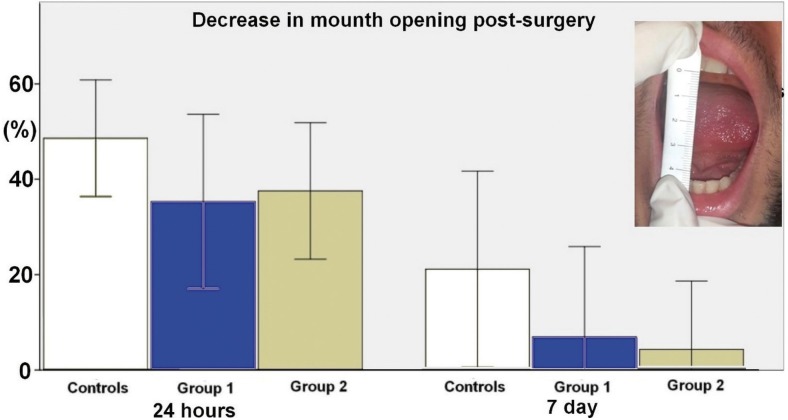
Shows the percentage of the decrease of mouth opening after surgery.

The trismus was evaluated measuring the interincisal opening, the maximal opening between the right maxillary and right mandibular central incisors before surgery (Fig. 3). Postoperative trismus was measured as a percentage of the decrease in mouth opening.

- Statistical analysis

Statistical analysis was performed using SPSS for Windows version 21 (IBM SPSS Inc., Chicago, IL, USA). Analysis of variance (ANOVA) and the Bonferroni/Dunn test were used to compare the parameters analyzed in the study. Data were analyzed using linear regression and descriptive statistics. The significance threshold was set at 0.05.

## Results

Lower scores in all studied parameters characterized both laser treated groups.

Figure 1 shows the visual analog scale (VAS) for pain referred by patients. The mean pain score at 24 hours was significantly higher in controls (7.80±1.84) respect group 1 (3.70±2.30) and 2 (2.26±0.90) at 24 hours post-surgery. Statistically significant differences have been found between controls and group 1 (*p*-value=0.012 S) and group 2 (*p*-value= 0.002 S). All patients referred no pain at 7 days after surgery. The level of significance was set at 0.05.

The amount of doses of 80 mg Ketoprofen assumed by the patients in the first 24 hours is shown in figure 1B. Group 2 referred to having assumed a lower quantity of Ketoprofen (0.52 times), respect controls (2.00 times; *p*-value<0.001 S) and group 1 (1.83 times; *p*-value<0.001 S).

Figure 2 shows the percentage (%) of increase in facial width after surgery. In the first postoperative day, the average percentage increase in facial width in the control group was 12.53±5.00; in group 1, it was 4.35±1.93 and group 2 was 6.17±5.23. Statistically significant differences have been found between controls and group 1 (*p*-value < 0.001 S) and 2 (*p*-value= 0.004 S). On the 7th postoperative day, the same parameter returned quite similar to the preoperative evaluation.

Figure 3 shows the percentage of the decrease of mouth opening after surgery. Results at 24h post-surgery were 50.02±12.23, 34.51±19.45 and 36.83±14.45 for what concerning controls, group 1 and 2, respectively. Although there were no significant differences between the treatment groups with regard to reduction in mouth opening, the “trismus” scores of group 1 and 2 were lower respect controls. At the 7th day after surgery a residual trismus was recorded especially in controls (13.04%±12.00), on the contrary the percentage of the decrease in mouth opening was minimal in other groups (group 1, 4.50±11.47 and group 2, 6.80±11.60). No statistically significant differences were recorded for this parameter at 24 hours and at 7 days.

## Discussion

A retrospective study, to verify the better peri-operatory protocol that was associated with minor complications, after the removal of impacted third molars, has been conducted.

We have found no studies in literature that analyzed the effects of pre-operatory LLLT on post-surgery complications after third molar removal.

Results have confirmed that a double dose of LLLT, one immediately before and another after the surgery, is effective in reducing the perceived pain of patients and edema at 24 h. We have found no statistically significant differences between the group irradiated also in the pre-surgery phase (group 2) respect that irradiated only after the extraction (group 1), for the analyzed parameters. However, these data gain more importance if we consider that the need of Ketoprofen assumption in the first 24 hours was statistically significant lower in group 2 respect controls and group 1.

Our results are in accordance with Koszowski *et al.* that reported the comparison of analgesic effect of magnetic and laser stimulation before oral procedures. Laser stimulation and alternating magnetic field applied directly before oral surgery were-shown to be effective as analgesic agents to decrease intra- and postoperative sensations (17).

Uspenskiĭ *et al.* used low intensity laser light, (wavelength 675 mm) as preoperative preparation in patients with surgical diseases of the lungs. He found many positive effects like antibacterial activity, reduction of endoscopic and morphological features of inflammation of the bronchial mucosa, stimulation of local immunity, the improvement of the respiratory system functional, positive dynamics of clinical status (a decrease of cough, dispnea, quantity of mucus discharge and haemoptysis) (18).

A possible explanation of analgesic effect of LLLT is the ability to modulate several signaling pathways and physiologic mechanisms involved in analgesia, like the increase of β-endorphin levels (β-ep) and the modulation of biochemicals related to pain, including substance P (SP), tumor necrosis factor-α (TNF-α), and cyclooxygenase-2 (COX-2) (19-21). Animal studies indicate that preoperative LLLT can act locally to prevent ischemic muscle damage by decreasing the activity of CK and the re-release of ROS, while increasing the levels of antioxidants and heat shock proteins (22).

For what concerning edema both irradiated groups were characterized by lower increase in facial width in the first 24 hours with statistically significant differences respect controls. The effect of LLLT in reducing post-operative edema is a direct consequence of the activation of lymphatic flow, and blood supply (23,24). There were no additive effects in group 2 respect group 1 for what concerning facial edema. A possible explanation could be a latency time during which an additional laser irradiation is not able to induce additive effects on blood and lymphatic circulation. Indeed it has been shown that LLLT promotes the rapid increase in the number and diameter of the capillaries within the first hours until the peak at the twelfth hour after irradiation with low-level lasers and the subsequent decrease to near-normal values (25).

The percentage of decrease in mouth opening post-surgery seem to be relatively influenced by LLLT. Both group 1 and 2 are characterized by a lower percentage at 24 hours respect controls, but no statistically significant differences have been found. The same results have been recorded at 7 days and the preoperative irradiation in adjunction to the postoperative one seems to permit a faster recovery of the interincisal opening. The reduction of this parameter is a direct consequence of trismus that derive from inflammation after surgical procedures but it is also caused by the persistent permanence of the patient with opened mouth which fatigues the elevator muscles of the jaw and temporo-mandibular joint (TMJ).

LLLT is reported to induce muscle relaxation (26). In particular, some researchers have reported that applying the laser on muscles before the fatigue-inducing exercise provides more satisfactory reduction of fatigue (27). However, in this study only masseter and medial pterygoid muscle has been irradiated. For this reason in order to have greater effects on interincisal opening also TMJ and other elevators muscles should be irradiated.

## Conclusion

Results confirm the usefulness of low-level laser therapy in reducing post-surgery complications of pain, edema and trismus.

A preoperative irradiation immediately before and another after the extraction seems to increase the analgesic effect of LLLT and it was connected with a lower necessity of NSAIDs in the first 24 hours after surgery. Trismus and edema are reduced in both laser treated groups; however, the preoperative LLLT seems not to confer additional benefits respect post-operatory irradiation.

Results suggest that LLLT could represent a viable tool for the control of pain in those patients for which pharmacological treatment is be contraindicated.

## References

[1] Piecuch JF (2012). What strategies are helpful in the operative management of third molars?. J Oral Maxillofac Surg.

[2] Kim K, Brar P, Jakubowski J, Kaltman S, Lopez E (2009). The use of corticosteroids and nonsteroidal antiinflammatory medication for the management of pain and inflammation after third molar surgery: a review of the literature. Oral Surg Oral Med Oral Pathol Oral Radiol Endod.

[3] D'Ercole S, Spoto G, Trentini P, Tripodi D, Petrini M (2016). In vitro inactivation of Enterococcus faecalis with a LED device. J Photochem Photobiol B.

[4] Chow R, Armati P, Laakso EL, Bjordal JM, Baxter GD (2011). Inhibitory effects of laser irradiation on peripheral mammalian nerves and relevance to analgesic effects: a systematic review. Photomed Laser Surg.

[5] Oliveira Sierra S, Melo Deana A, Mesquita Ferrari RA, Maia Albarello P, Bussadori SK, Santos Fernandes KP (2013). Effect of low-level laser therapy on the post-surgical inflammatory process after third molar removal: study protocol for a double-blind randomized controlled trial. Trials.

[6] Asnaashari M, Safavi N (2013). Application of Low level Lasers in Dentistry (Endodontic). J Lasers Med Sci.

[7] Elbay ÜŞ, Tak Ö, Elbay M, Uğurluel C, Kaya C (2016). Efficacy of Low-Level Laser Therapy in the Management of Postoperative Pain in Children After Primary Teeth Extraction: A Randomized Clinical Trial. Photomed Laser Surg.

[8] Cotler HB, Chow RT, Hamblin MR, Carroll J (2015). The Use of Low Level Laser Therapy (LLLT) For Musculoskeletal Pain. MOJ Orthop Rheumatol.

[9] Baltzer AW, Ostapczuk MS, Stosch D (2016). Positive effects of low level laser therapy (LLLT) on Bouchard's and Heberden's osteoarthritis. Lasers Surg Med.

[10] Stasinopoulos D, Papadopoulos K, Lamnisos D, Stergioulas A (2016). LLLT for the management of patients with ankylosing spondylitis. Lasers Med Sci.

[11] Ferrante M, Petrini M, Trentini P, Perfetti G, Spoto G (2013). Effect of low-level laser therapy after extraction of impacted lower third molars. Lasers Med Sci.

[12] Avci P, Gupta A, Sadasivam M, Vecchio D, Pam Z, Pam N (2013). Low-level laser (light) therapy (LLLT) in skin: stimulating, healing, restoring. Semin Cutan Med Surg.

[13] He WL, Yu FY, Li CJ, Pan J, Zhuang R, Duan PJ (2015). A systematic review and meta-analysis on the efficacy of low-level laser therapy in the management of complication after mandibular third molar surgery. Lasers Med Sci.

[14] Rana MV, Desai R, Tran L, Davis D (2016). Perioperative Pain Control in the Ambulatory Setting. Curr Pain Headache Rep.

[15] Neĭmark AI, Muzalevskaia NI (2000). [Low-intensity laser radiation in preoperative preparation of patients with benign prostatic hyperplasia]. Urologiia.

[16] Almendros-Marqués N, Berini-Aytés L, Gay-Escoda C (2006). Influence of lower third molar position on the incidence of preoperative complications. Oral Surg Oral Med Oral Pathol Oral Radiol Endod.

[17] Koszowski R, Smieszek-Wilczewska J, Dawiec G (2006). Comparison of analgesic effect of magnetic and laser stimulation before oral surgery procedures. Wiad Lek.

[18] Uspenskiĭ LV, Chistov LV, Kogan EA, Loshchenov VB, Ablitsov IuA, Rybin VK (2000). Endobronchial laser therapy in complex preoperative preparation of patients with lung diseases. Khirurgiia (Mosk).

[19] Camillo de Carvalho PT, Leal-Junior ECP, Alves ACA, De Melo Rambo CS, Sampaio LMM, Oliveira CS (2012). Effect of low-level laser therapy on pain, quality of life and sleep in patients with fibromyalgia: study protocol for a double-blinded randomized controlled trial. Trials.

[20] Gür A, Karakoç M, Nas K, Cevik R, Saraç J, Demir E (2002). Efficacy of low power laser therapy in fibromyalgia: a single-blind, placebo-controlled trial. Lasers Med Sci.

[21] Hsieh YL, Hong CZ, Chou LW, Yang SA, Yang CC (2015). Fluence-dependent effects of low-level laser therapy in myofascial trigger spots on modulation of biochemical associated with pain in a rabbit model. Lasers Med Sci.

[22] Rizzi CF, Mauriz JL, Freitas Corre^a DS, Moreira AJ, Zettler CG, Filippin LI (2006). Effects of low-level laser therapy (LLLT) on the nuclear (NF)- kappaB signaling pathway in traumatized muscle. Lasers Surg. Med.

[23] Tomaz de Magalhães M, Núñez SC, Kato IT, Ribeiro MS (2016). Light therapy modulates serotonin levels and blood flow in women with headache. A preliminary study. Exp Biol Med (Maywood).

[24] Smoot B, Chiavola-Larson L, Lee J, Manibusan H, Allen DD (2015). Effect of low-level laser therapy on pain and swelling in women with breast cancer-related lymphedema: a systematic review and meta-analysis. J Cancer Surviv.

[25] Ihsan FR (2005). Low-level laser therapy accelerates collateral circulation and enhances microcirculation. Photomed Laser Surg.

[26] Herpich CM, Leal-Junior ECP, Amaral AP, de Paiva TJ, dos Santos Glória IP, Garcia MBS (2014). Effects of phototherapy on muscle activity and pain in individuals with temporo-mandibular disorder: a study protocol for a randomized controlled trial. Trials.

[27] Leal Junior EC, Lopes–Martins RA, Frigo L, De Marchi T, Rossi RP, de Godoi V (2010). Effects of low-level laser therapy (LLLT) in the development of exercise-induced skeletal muscle fatigue and changes in biochemical markers related to post-exercise recovery. J Orthop Sports Phys. Ther.

